# PDLIM1 sustains DNA damage response by promoting chromatin relaxation and activating DNA–PK complex

**DOI:** 10.1016/j.gendis.2023.101139

**Published:** 2023-10-13

**Authors:** Yue Lang, Dexuan Gao, Lan Yu, Xiang-Xiang Zhang, Debabrata Saha, Benjamin P.C. Chen, Meng-Meng Gu, Zeng-Fu Shang

**Affiliations:** a Department of Nuclear Medicine, The Second Affiliated Hospital of Xuzhou Medical University, Xuzhou, Jiangsu 221000, China; b Department of Nuclear Medicine, The Affiliated Suzhou Hospital of Nanjing Medical University, Suzhou, Jiangsu 215002, China; c State Key Laboratory of Radiation Medicine and Protection, School of Radiation Medicine and Protection, Medical College of Soochow University, Collaborative Innovation Center of Radiation Medicine of Jiangsu Higher Education Institutions, Soochow University, Suzhou, Jiangsu 215123, China; d Department of Urology, Shandong Provincial Hospital Affiliated to Shandong First Medical University, Jinan, Shandong 250021, China; e Department of Radiation Oncology, University of Texas Southwestern Medical Center, Dallas, TX 75390, USA; f Department of Pathology, University of Texas Southwestern Medical Center, Dallas, TX 75390, USA

PDZ and LIM domain protein 1 (PDLIM1) is a cytoplasmic LIM protein that regulates cytoskeleton organization and functions as a platform to coordinate various cellular processes.^1^ Accumulated evidence suggests that PDLIM1 plays important roles in tumorigenesis, tumor progression, and response to cancer therapy. Consistently, our results demonstrated that silencing PDLIM1 significantly inhibited the migration and the invasion of HeLa cells compared with control cells (Fig. S1). However, the feasibility of targeting PDLIM1 as an effective strategy for radiotherapy remains to be determined. Here, we knocked down PDLIM1 while irradiating with ^60^Co γ-rays (IR), and impaired clonogenic survival was observed in PDLIM1-depleted HeLa cells (Fig. 1A, B). Furthermore, the heightened radiosensitivity prompted by PDLIM1 knockdown was effectively counteracted through the expression of siRNA-resistant PDLIM1 protein (Fig. S2A, B). To determine whether PDLIM1 affects DNA damage repair following IR exposure, we performed a neutral comet assay. PDLIM1 knockdown cells have significantly more unrepaired IR-induced DNA double strands breaks (DSBs) compared with control cells, as monitored by comet tail moment (Fig. 1C). There are two primary mechanisms for the restoration of DNA DSBs: homologous recombination (HR) and non-homologous end joining (NHEJ). Therefore, the quantitative assessment of NHEJ and HR efficiencies was conducted *in vivo* using a CRISPR/Cas9-mediated oligodeoxynuceotide (ODN) detection system.^2^ The loss of PDLIM1 significantly reduces both NHEJ and HR activity within HeLa cells (Fig. 1D). Subsequently, HeLa cells were subjected to IR, followed by immunofluorescence staining targeting γH2AX, which represents a prominent chromatin modification trigger by several phosphatidylinositol 3-kinase-related family members after DNA DSBs. Interestingly, the depletion of PDLIM1 substantially abrogated the formation of γH2AX after IR exposure (Fig. 1E). The recruitment of mediator of DNA damage checkpoint protein 1 (MDC1) by γH2AX is recognized as the initial step in which the site of a DSB undergoes preparation for the activation of DNA damage response (DDR). Immunofluorescence staining also revealed that IR-induced MDC1 foci formation was attenuated in PDLIM1-deficient cells at the early time points post-IR compared with control cells (Fig. 1F). Furthermore, the hypothesis proposing that the recruitment of DDR factors to DNA DSBs necessitates the presence of PDLIM1 was further substantiated by the observation that the localization of 53BP1 (Fig. 1G) and BRCA1 (Fig. S2C) — both critical factors in NHEJ and HR pathway, respectively — at DSBs sites was diminished in cells depleted of PDLIM1, as compared with the control cells.

**Figure 1:**
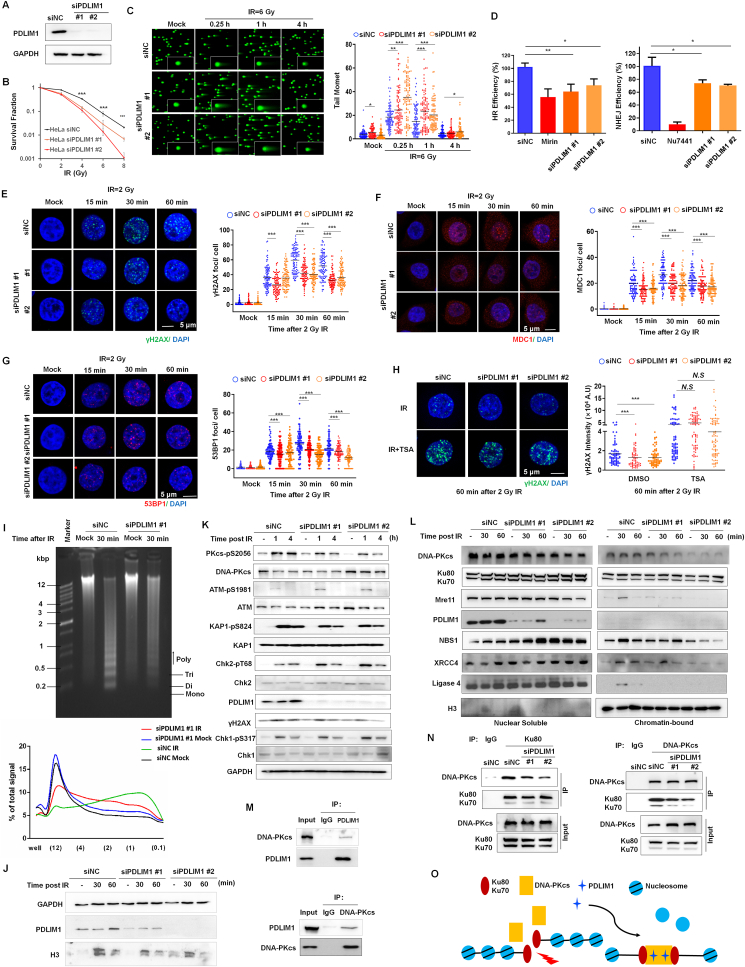
PDLIM1 sustains DNA damage response by promoting chromatin relaxation and activating the DNA–PK complex. **(A)** Immunoblot analysis of the siRNA-mediated suppression of PDLIM1 protein in HeLa cells. **(B)** Clonogenic cell survival curve of PDLIM1-depleted and control HeLa cells exposed to 2, 4, 6, or 8 Gy of γ-ray irradiation. Data are represented as mean ± standard deviation from three independent experiments. ^∗∗∗^*P* < 0.001. **(C)** Representative comet images of different groups of cells. The repair ability of DNA DSBs was measured (more than 100 cells were counted, three independent assays, ^∗∗^*P* < 0.01, ^∗∗∗^*P* < 0.001). **(D)** HeLa cells were transfected with either siRNA against PDLIM1 or control siRNA. 24 h later, cells were additionally transfected with 3 μg Cas9/sgHPRT plasmid and 20 pmol ssODN (left panel) or 25 pmol dsODN (right panel). Real-time PCR analysis was conducted to assess the efficiency of HR (left panel) and NHEJ (right panel) repair in different cell groups, Mirin and Nu7441 were used as positive control of HR and NEHJ repair. **(E**–**G)** Representative immunofluorescence images showing γH2AX (E), MDC1 (F), and 53BP1 (G) foci in either PDLIM1-silenced or control HeLa cells treated with 2 Gy IR (scale bar = 5 μm). The number of γH2AX, MDC1, and 53BP1 foci per cell was presented at the indicated time points following IR (data were generated from three independent experiments. ^∗^*P* < 0.05, ^∗∗∗^*P* < 0.001). **(H)** PDLIM1-depleted and control HeLa cells were exposed to 2 Gy IR following the treatment with or without 100 nM trichostatin A (TSA) for 2 h and stained with γH2AX. The intensity of γH2AX staining per cell was displayed (data were generated from three independent experiments. ^∗∗∗^*P* < 0.001; *N.S* represents non-significance). **(I)** IR-induced chromatin relaxation is attenuated in PDLIM1-depleted HeLa cells. The PDLIM1-depleted and control HeLa cells were mock-treated or irradiated with a dose of 20 Gy and allowed to recover for 30 min. Chromatin decondensation was determined by examining micronuclease (MNase) digestion. The lower panel showed a quantified signal as the percent of the total for each lane across a distance from the well to the end of the gel. **(J)** The PDLIM1-depleted and control HeLa cells were mock-treated or irradiated with 10 Gy γ-ray, cells were fractionated in 0.8 M NaCl, and released histones were detected by immunoblotting. **(K)** Control and siPDLIM1-transfected HeLa cells were exposed to 10 Gy γ-ray and harvested at the indicated time points. Whole cell lysates were subjected to immunoblotting analysis. **(L)** Control and siPDLIM1-transfected HeLa cells were mock-treated or irradiated with 10 Gy γ-ray and allowed to recover for 30 min and 1 h. Subsequently, the soluble nuclear fraction and the chromatin-bound fraction were isolated for immunoblotting analysis. **(M)** HeLa cell lysates were immunoprecipitated with anti-DNA-PKcs, anti-PDLIM1 antibodies, or mouse IgG followed by immunoblotting analysis to detect PDLIM1 and DNA-PKcs levels. **(N)** Control and siPDLIM1-transfected HeLa cell lysates were immunoprecipitated with anti-DNA-PKcs, anti-Ku80 antibodies, or mouse IgG followed by immunoblotting analysis to detect Ku80/Ku70 and DNA-PKcs levels. **(O)** This schematic shows that PDLIM1 promotes chromatin relaxation and DNA damage response in response to IR by regulating the stability of the DNA–PK complex.

Eukaryotic DNA is organized into compact chromatin. Therefore, chromatin remodeling plays a critical role in facilitating DNA access, regulating the activity of repair proteins, and mediating signaling transduction in response to DNA DSBs. Next, we examined whether PDLIM1's function could make chromatin permissive for DDR activation. We treated HeLa cells with low concentration (100 nM) of the HDAC inhibitor trichostatin A for 5 h. Trichostatin A treatment led to an enhancement of γH2AX formation in HeLa cells, while also mitigating the differences arising from PDLIM1 depletion (Fig. 1H). This suggests that PDLIM1's contribution to DDR progression may stem from its potential role in chromatin relaxation. To assess the impact of PDLIM1 on chromatin accessibility, chromatin digestion using micrococcal nuclease was performed after IR. IR-induced DNA damage notably heightened the susceptibility of chromatin to micrococcal nuclease digestion in control HeLa cells 30 min after IR. Still, this effect was significantly diminished in PDLIM1-depleted cells (Fig. 1I). Histone-DNA interaction is highly stable, and the release of histone from chromatin typically occurs when NaCl concentrations exceed 1.5 M. Histones can be preferentially released in the context of damaged chromatin due to enhanced nucleosome dynamics. As shown in Figure 1J, H3 was significantly eluted from IR-treated HeLa cells at 0.8 M NaCl, but not from untreated cells at the same salt concentration of salt. However, the absence of PDLIM1 resulted in a decrease in the elutability of H3 after DNA damage (Fig. 1J). Nuclear actin has been implicated in a diverse range of DNA-related processes, including chromatin remodeling and DNA damage repair.^3^ As an adaptor and partner of actin, it remains uncertain whether the nuclear function of PDLIM1 depends on its interaction with actin. Here, we utilized jasplakinolide, a compound that binds and stabilizes actin dimer, and swinholide A, a substance that induces actin deplymerization, to treat HeLa cells in combination with IR exposure. Our finding revealed that treatment with both jasplakinolide and swinholide A led to an increase in γH2AX formation following IR. However, these agents did not eliminate the differences in γH2AX formation between PDLIM1-deficient and control cells (Fig. S3).

DNA-PKcs, a member of the phosphatidylinositol 3-kinase-related kinase family, primarily participates in the NHEJ repair pathway. A recent study has unveiled the critical role of DNA-PKcs in chromatin decondensation near DNA damage sites.^4^ PDLIM1-depleted and control HeLa cells were exposed to varying durations of IR, and the activation of DNA-PKcs was assessed by monitoring its autophosphorylation at Ser2056. After IR treatment, we noted a reduced level of DNA-PKcs autophosphorylation in PDLIM1-knockdown cells compared with the control cells (Fig. 1K). Additionally, we observed impaired phosphorylation of KRAB domain-associated protein 1 at Ser824, a known contributor to heterochromatin relaxation, in PDLIM1-depleted HeLa cells compared with the control cells (Fig. 1K). However, PDLIM1-depleted HeLa cells exhibited heightened activation of the ATM-Chk2 signaling pathway, potentially suggesting the presence of more severe DNA damage compared with the control cells (Fig. 1K). To assess the impact of PDLIM1 on the recruitment of DDR machinery, chromatin fractions were isolated from PDLIM1-depleted and control HeLa cells. The depletion of PDLIM1 significantly diminished the chromatin recruitment of Mre11, NBS1, Ligase 4, XRCC4, and DNA-PKcs, but not Ku80/Ku70 (Fig. 1L). Although PDLIM1 was not detected in the chromatin fraction, it was notably present in the nuclear soluble fraction (Fig. 1L). This observation was further corroborated by immunofluorescence staining, which demonstrated the nuclear presence of PDLIM1 (Fig. S4). Next, the interaction between PDLIM1 and DNA-PKcs was assessed in HeLa cells using a co-immunoprecipitate assay (Fig. 1M), and the depletion of PDLIM1 markedly hindered the interaction of DNA-PKcs with its partner Ku70/Ku80 (Fig. 1N). As depicted in Figure S5B, the LIM domain of PDLIM1 is responsible for its interaction with DNA-PKcs. The GST pull-down assay results demonstrated that PDLIM1 could indeed interact directly with Ku80 and the T2609 cluster of DNA-PKcs (Fig. S5C, D). DNA-PKcs has also been demonstrated to regulate the mitotic process of both normal and IR-damaged cells by maintaining the stability of the spindle structure.^5^ Although the precise localization of PDLIM1 on the spindle structure was not observed (data not shown), the absence of PDLIM1 resulted in a significant increase in aberrant mitosis and a higher occurrence of multinucleated cells after exposure to IR at 24 h post-IR (Fig. S6).

Overall, our study has unveiled a novel role for PDLIM1 in the regulation of DNA damage response and chromatin remodeling through its interaction with the DNA–PK complex, highlighting its critical importance in preserving genome integrity (Fig. 1O). Investigating whether PDLIM1 influences the retention of DNA-PKcs at the damaged site presents an intriguing avenue for exploration. These findings may provide valuable insights into the development of innovative therapeutic strategies targeting these vulnerabilities in cancer cells.

## Author contributions

Yue Lang: Performing the experiments; Data curation; Study concept; Supervision. Lan Yu: Data curation; Manuscript writing; Dexuan Gao and Xiang–Xiang Zhang: Performing the experiments; Data analysis. Debabrata Saha: Study concept. Benjamin P.C Chen: Study concept; Critical design. Meng–Meng Gu: Study concept; Supervision. Zeng-Fu Shang: Study concept; Critical design; Supervision; Validation.

## Conflict of interests

The authors declare no conflict of interests.

## Funding

The research was funded by grants from the 10.13039/501100001809National Natural Science Foundation of China (No. 82003226 and 81972964), the 10.13039/501100012246Priority Academic Program Development of Jiangsu Higher Education Institutions (10.13039/501100012246PAPD), and the 10.13039/501100007129Natural Science Foundation of Shandong Province (ZR2021MH085).
