# Supplementary material for: PDLIM1 sustains DNA damage response by promoting chromatin relaxation and activating DNA–PK complex
Source: Genes Dis. 2023 Oct 13;11(6):101139. doi: 10.1016/j.gendis.2023.101139 (PMC11283213; doi:10.1016/j.gendis.2023.101139)
Supplement: Multimedia component 1 [file mmc1.docx]

**PDLIM1 sustains DNA damage response through promoting chromatin relaxation and activating DNA-PK complex**

**Supplementary Materials**

**Materials and methods**

**1. Cell culture and irradiation**

HeLa cells were maintained in Dulbecco’s modified Eagle’s medium (Invitrogen, Carlsbad, CA, USA) supplemented with 10% FBS (HyClone, Hudson, NH, USA), 100 units/mL penicillin, and 100 µg/mL streptomycin in a humidified incubator at 37 °C in 5% CO_2_. A ^60^Co γ-ray source at the dose rate of 2 Gy/min was used to irradiate the cells, and the cells were harvested at an indicated time of post-irradiation culture and then subjected to further experiments.

**2. siRNAs and plasmids**

Duplex siRNAs were synthesized (Invitrogen, Carlsbad, CA, USA) based on experimentally validated target sequences for PDLIM1 siRNA #1 (5′-UCAGAAGUUGCCUAUGUGUTT-3′), siRNA #22 (5′-CUUGGUUAAUUGACUCACATT-3′) or control siRNA (5′-CTGGACTTCCAGAAGAACA-3′) . siRNAs were transfected into HeLa cells using Lipofectamine 3000 (Invitrogen, Carlsbad, CA, USA) according to the manufacturer's protocol. PDLIM1 full length and truncation constructs was cloned into a pCMV-tag2B vector with a N-terminal FLAG tagged expression plasmids.

**3. Antibodies and immunoprecipitation**

The following primary antibodies were used: rabbit anti-DNA-PKcs-pSer2056, anti-PDLIM1 (abcam, Cambridge, MA, USA); rabbit anti-DNA-PKcs, mouse anti-Ku80, anti-Brca1, goat anti-Ku70, control mouse IgG and rabbit IgG (Santa Cruz Biotechnology Inc, Dallas, TX, USA); rabbit anti-KAP1-pS824, anti-KAP1 (Bethyl Laboratories, Montgomery, TX, USA); mouse anti-ATM, rabbit anti-53BP1, anti-ATM-pSer1981, anti-Chk2-pThr68, anti-Chk2, anti-histone 3 (Cell Signaling Technology Danvers, MA, USA); mouse anti-phospho–histone 2AX (Ser-139) (Millipore, Bellerica, MA, USA); rabbit anti-MDC1, anti-Mre11, anti-NBS1 (Novus Biologicals, Centennial, CO, USA); mouse anti-GAPDH, anti-Flag (Sigma-Aldrich, St. Louis, MO, USA). Secondary antibodies used for immunofluorescence were Alexa Fluor 488 goat anti-mouse IgG, Alexa Fluor 488 goat anti–rabbit IgG, Alexa Fluor 488 goat anti–human IgG, Alexa Fluor 568 goat anti–mouse IgG and Alexa Fluor 568 goat anti–rabbit IgG (Invitrogen Carlsbad, CA, USA).

HeLa cell lysates were incubated with anti-DNA-PKcs, PDLIM1 antibodies and protein A/G sepharose overnight. The sepharose beads were washed with lysis buffer three times and resuspended in SDS-PAGE loading buffer for immunoblot analysis using anti-PDLIM1, DNA-PKcs antibodies. Flag-fused full length PDLIM1 and PDLIM1 truncations were transfected into HeLa cells. Cells were harvested and cell lysates were incubated with anti-Flag-M2 magnetic beads for overnight. The magnetic beads were washed with lysis buffer three times and resuspended in SDS-PAGE loading buffer for immunoblot analysis using anti-Flag, DNA-PKcs and Ku80 antibodies.

**4. Immunofluorescence**

For immunofluorescence analysis, cells were plated on 35-mm dishes with coverslips, fixed in 4% paraformaldehyde/PBS for 30 min at indicated time points after irradiation, permeabilized in 0.5% Triton X-100/PBS for 15 min, and blocked in 5% bovine serum albumin for 30 min. The samples were incubated with anti-γ-H2AX (1:1000), anti-53BP1 (1:1000), anti-Brca1 (1:500), anti-MDC1 (1:200), anti-α-tubulin (1:1000), anti-CREST (1:2000) antibodies for 4 h, washed three times in phosphate buffered saline (PBS) (10 min each) and incubated with rhodamine red-conjugated goat anti-mouse secondary antibodies (1:1000) for 1 h. The cells were washed in PBS three times (10 min each) and mounted using VECTASHIELD with 4′, 6-diamidino-2-phenylindole (DAPI) (Vector Laboratories, Burlingame, CA, USA). The actin was stained by phalloidine. Images were taken under a fluorescence microscope (Axio Imager M2, Carl Zeiss, Thornwood, NY, USA) with AxioVision SE64 Rel.4.8 software (Carl Zeiss, Thornwood, NY, USA). For Brca1 staining, cells were pre-extracted for 10 min at room temperature with CSK buffer (10 mM Pipes, pH 7.0, 100 mM NaCl, 300 mM sucrose, and 3 mM MgCl_2_) containing 0.7% Triton X-100.

**5. Subcellular fractionation**

The association of DDR proteins to chromatin following IR-induced DNA damage was examined using the Thermo Fisher Subcellular Protein Fractionation Kit (Thermo Fisher, Waltham, MA USA ). Briefly, the PDLIM1 knockdown and control HeLa cells were mock-treated or irradiated with 10 Gy IR and allowed to recover for 30 min and 1 h. Next, the cells were harvested after trypsinization and processed with the Thermo Fisher Subcellular Protein Fractionation Kit according to the manufacturer's instructions. The protein concentration of each sample was measured using a Pierce BCA Protein Assay kit (Thermo Fisher) and 30 μg protein of each sample was separated via SDS-PAGE, and then transferred to a PVDF membrane for western blotting analysis using the protocol outlined above.

**6. Comet Assay for DNA Double-Strand Breaks**

HeLa cells were transfected with siRNA against PDLIM1. After 24 hours, cells were irradiated with 6 Gy γ-ray, A duration of indicated time points, the cells were mixed with low melting point agarose, spread on a comet assay slide. Those slides were left into 4 °C for drying, and incubated with neutral lysis buffer and subjected to electrophoresis. Cells were stained with SYBR Green I, and comet tails were visualized using a confocal microscope (FV1200, Olympus, Tokyo, Japan). Experiments were performed at least three times for each sample.

**7 NHEJ and HR efficiency detection**

The CRISPR/Cas9 plasmid used in this study was provided by Dr. Jiahua Yu from School of Radiation Medicine Protection, Medical College of Soochow University, Suzhou, China. The ssODN and dsODN sequences were synthesized by Sangon (Shanghai, China). For the experimental procedure, HeLa cells were seeded in a 6-well plate containing 10% fetal bovine serum. After 24 hours, siRNA was transfect into HeLa cells using the lipofectamine 3000 (Invitrogen, Carlsbad, CA, USA). Subsequently, the CRISPR/Cas9 plasmid (2.5 μg) was co-transfected with ssODN (20 pmol) for HR detection, or dsODN (25 pmol) for NHEJ efficiency detection after 24 hours. The DNA-PKcs inhibitor Nu7441 and Mre11 inhibitor Mirin (Selleck, Houston, TX, USA) were used as positive control to assess the reduction in the levels of NHEJ and HR repair. Genomic DNA from the cells was extracted on alternate days using the Genomic DNA Kit (Tiangen, Beijing, China). The real-time PCR reaction mixture included 100 ng of genomic DNA, 200 nM forward and reverse primers (Sangon, Shanghai, China), and SYBR Green Mixture (Tiangen, Beijing, China), making a final volume of 20 μl. For the NHEJ system, the forward primer sequence was: 5′-AGGTTATGACCTTGATTTATTTTGCA-3′, and the reverse primer sequence was: 5′-AACAGCTGCTGATGTTTGAAATTAA-3′. The primer used for the HR system was the same as the one used for the NHEJ system. The real-time PCR reaction was performed using the ViiA 7 Real-time PCR System (Applied Biosystems, Foster City, CA).

**8 Micronuclease (MNase) digestion assay**

In 10 cm dishes, PDLIM1-depleted and control HeLa cells were grown to 90% confluency and were mock-treated or irradiated with a γ-ray dose of 20 Gy and allowed to recover for 30 min. Cells were washed with ice-cold PBS and then resuspended in ice-cold hypotonic Hank's balanced salt solution (HBSS) buffer (340 mM sucrose, 15 mM Tris, pH 7.5, 15 mM NaCl, 60 mM KCl, 10 mM DTT, 0.15 mM spermine, 0.5 mM spermidine and 0.5% Triton X-100) and allowed to incubate on ice for 10 min with periodic vortexing. The nuclei were pelleted via centrifugation at 11,000 rpm for 5 min at 4°C, washed with HBSS buffer, resuspended in 1:1 HBSS/glycerol, and stored at −20°C overnight. Subsequently, the nuclei were centrifuged and resuspended in 75 μl MNase digestion buffer (250 mM sucrose, 15 mM Tris, pH 7.5, 15 mM NaCl, 60 mM KCl, 0.5 mM DTT and 1 mM CaCl2). The MNase digestion was performed at 25°C after adding 1.7 μl 1 U/ml MNase to 75 μl of resuspended nuclei for 3 min and stopped by adding 1.5 μl of 0.5 M EDTA and 8 μl of 5% SDS with 1 mg/ml Proteinase K (Sigma-Aldrich), and each sample was incubated at 37°C for 30 min. The DNA was extracted with phenol/chloroform/isoamyl-alcohol and precipitated with isopropanol and sodium acetate. After measuring the DNA concentration, 2 μg of each sample was separated on a 1.2% agarose gel electrophoresed in Tris-acetate EDTA buffer, stained with ethidium bromide, and visualized via the Bio-Rad ChemiDoc™ MP Imaging System. The DNA band intensity of each sample were quantified with Image J version 1.51 and normalized to total volume of the whole lane.

**9 Nucleosome stability assay**

Cells were harvested and washed twice in ice-cold PBS by centrifugation at 1,000 rpm. Cell pellet was resuspended completely in 500 µl buffer A (20 mM Hepes, pH 7.9, 0.5 mM DTT, 1 mM PMSF, 1.5 mM MgCl2, and 0.1% Triton) containing 0.8 M NaCl. Cells were incubated for 40 min at 4°C with constant agitation. Samples were then centrifuged at 100,000 g (Ultracentrifuge; Beckman Coulter) for 20 min, and the supernatant, containing released histones, retained for further analysis. In some experiments, cells were first incubated in buffer A with 0.15 M NaCl to remove cell membrane and cytosolic contents. The nuclear fraction was then collected by centrifugation and the nuclear pellet resuspended in buffer A plus 0.8 M NaCl and processed as described above. Essentially identical results were obtained using both methods.

**10 Flag-fused PDLIM1 purification and GST pull down assay**

For purification of 3×FLAG-tagged PDLIM1 from HeLa cells, the plasmid was transfected into HeLa cells. The cells expressing Flag-PDLIM1 were suspended briefly in lysis buffer 3 (50 mM HEPES 7.4, 500 mM NaCl, 0.5% NP-40, 10% Glycerol, 1× phosphatase inhibitor cocktail, 1× protease inhibitor cocktail) and sonicated on ice. Cell lysates were incubated with M2-FLAG beads (Sigma, St. Louis, MO, USA). Flag-tagged PDLIM1 were treated with lambda phosphatase (γPP) (Sigma, St. Louis, MO, USA). Protein-bound M2-Flag beads were washed with washing buffer 3 (50 mM HEPES pH 7.4, 1 M NaCl, 0.5% NP-40, 10% Glycerol, 1× phosphatase inhibitor cocktail, 1× protease inhibitor cocktail), lysis buffer 3, and washing buffer 4 (50 mM HEPES 7.4, 150 mM NaCl, 0.5% NP-40, 10% Glycerol), washing buffer 5 (50 mM HEPES 7.4, 150 mM NaCl, 10% Glycerol), and washing buffer 6 (50 mM HEPES 7.4, 80 mM KCl, 10% Glycerol). Finally, proteins were eluted with washing buffer 6 containing 250 µg mL^−1^ 3×FLAG peptides. Protein purity was examined by Coomassie blue gel staining after SDS-PAGE, and the concentration was measured with Bio-Rad Protein Assay Kit 1.

For glutathione S-transferase (GST) pull-down assay, GST–Ku70, -Ku80 and -DNA-PKcs fusion proteins were incubated with glutathione-sepharose beads (GE Healthcare Life Sciences, Piscataway, NJ) and then recombinant Flag-PDLIM1 proteins followed by immunoblot analysis.

**11 Statistical analysis**

Data are presented as the mean ± SD of at least three independent experiments. The results were tested for significance using the unpaired Student's t-test (^**^P < 0.01 and ^*^P < 0.05 were considered significant).


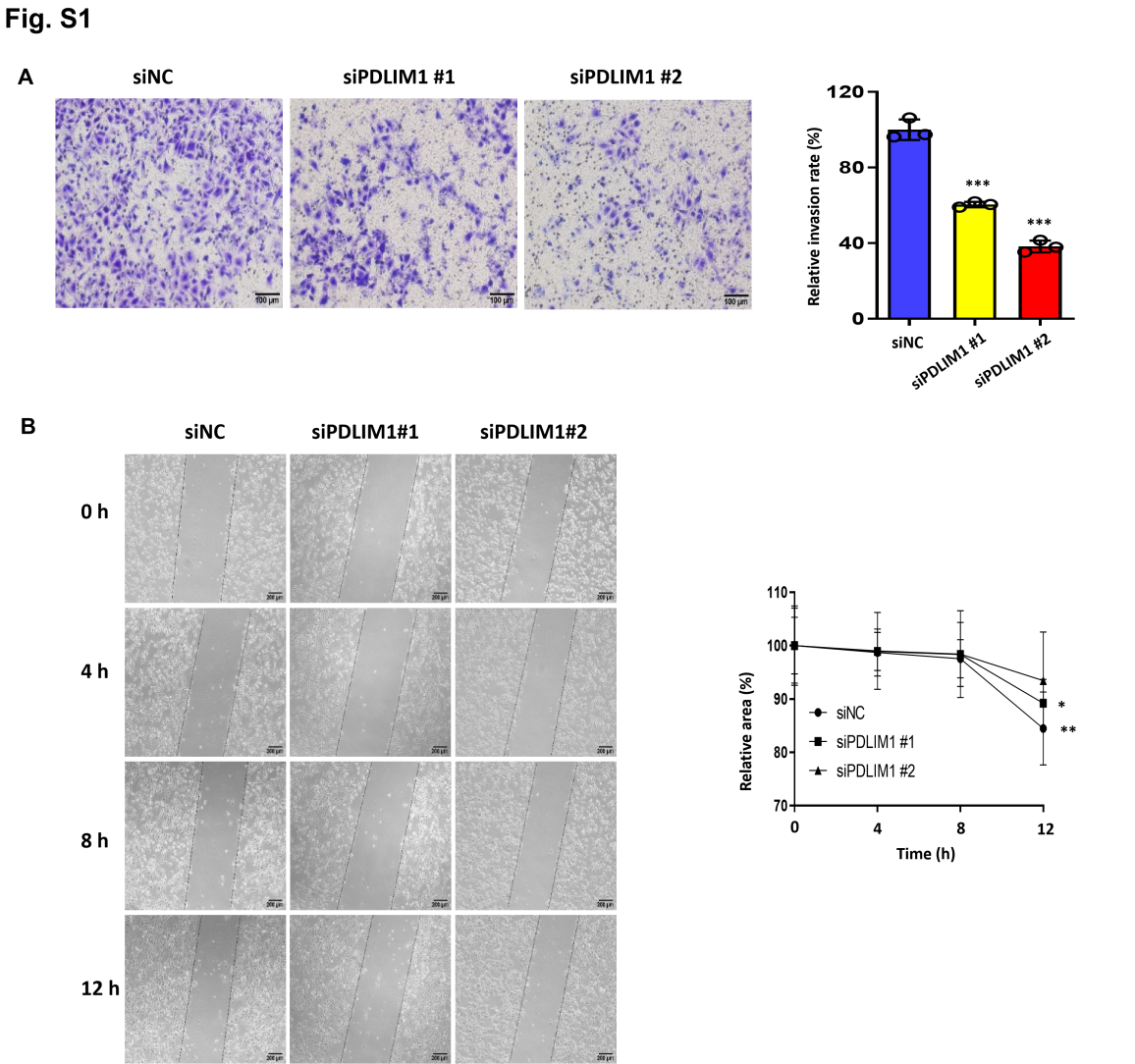


**Supplemental Figure 1.** PDLIM1 promotes HeLa invasion and cell migration. (**A**) Cell invasion of HeLa cells was assessed after transfection with PDLIM1-specific siRNA (siPDLIM1) #1, #2 or non-targeting control siRNA (siNC), following the procedure described in the Materials and Methods. Left panel: Representative images of the invading cells are shown (200× magnification). Right panel: Histogram of invading cells per field of transfected HeLa cells determined by counting the invading cells in 5 random fields. Data were presented as mean ± SD from 3 independent experiments (****p* < 0.001). (**B**) Cell migration of HeLa cells was evaluated following transfection with PDLIM1-specific siRNA (siPDLIM1) #1, #2 or non-targeting control siRNA (siNC), according to the methodology described in the Materials and Methods. Left panel: Representative images of the migrated cells are shown. Right panel: Histogram displaying the relative scratched area of transfected HeLa cells (**p* < 0.05, ***p* < 0.01).


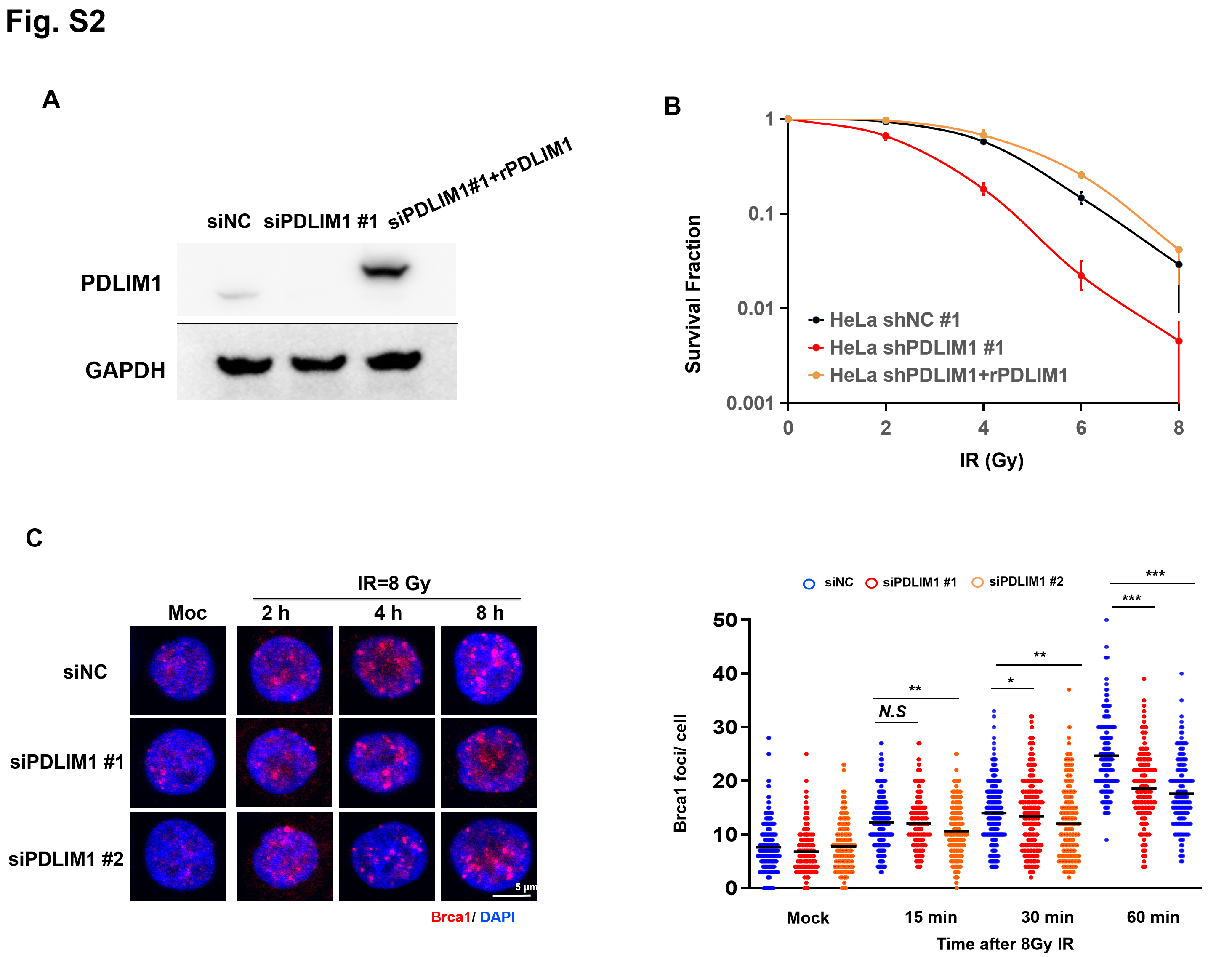


**Supplemental Figure 2.** (**A**) Immunoblot analysis of the siRNA-mediated PDLIM1 suppression and re-expression of siRNA-resistant PDLIM1 (rPDLIM1) construct in HeLa cells. (**B**) Clonogenic cell survival curve of PDLIM1-depleted, control and PDLIM1 reintroduced HeLa cells exposed to 2, 4, 6 or 8 Gy of γ-ray irradiation. (**C**) Representative IF images showing Brca1 foci in either PDLIM1-silenced or control HeLa cells treated with 8 Gy IR (scale bar = 5 μm). The number of Brca1 foci per cell was presented at the indicated time points following IR (data were generated from three independent experiments. * *p* < 0.05, ** *p* < 0.01, *** *p* < 0.001).


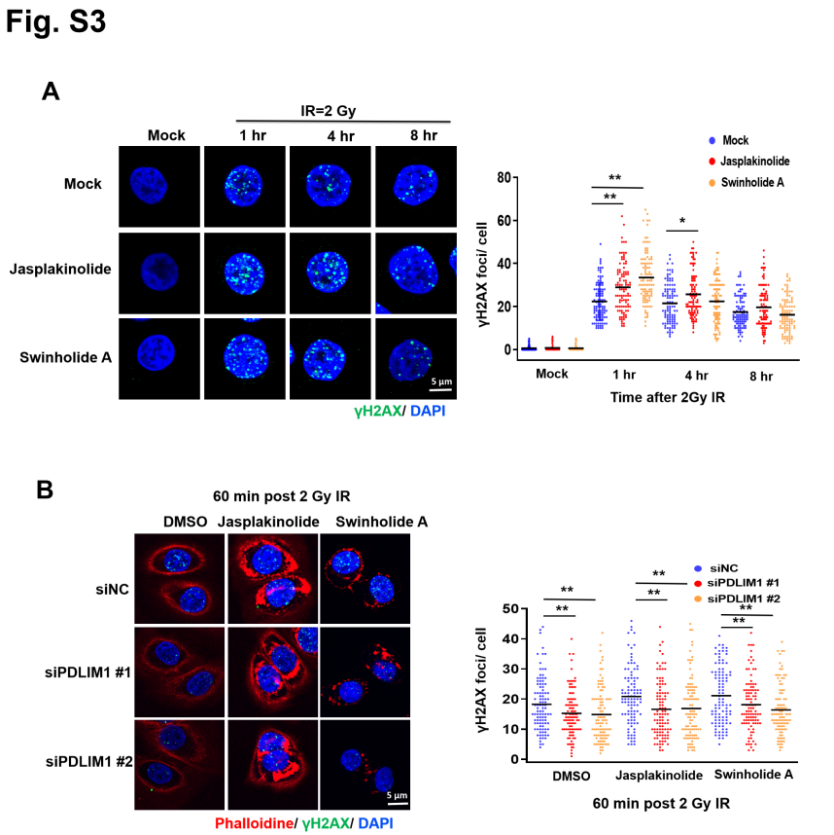


**Supplemental Figure 3.** (**A**) HeLa cells were pre-treated with Jasplakinolide and Swinholide A for 2 hours, and exposed to 2 Gy irradiation. Representative IF images showing γH2AX foci in either agents-treated or mock HeLa cells exposed to 2 Gy IR (scale bar = 5 μm). The number of γH2AX foci per cell was presented at the indicated time points following IR (data were generated from three independent experiments. * *p* < 0.05, ** *p* < 0.01). (**B**) PDLIM1-depleted and control HeLa cells were pre-treated with Jasplakinolide and Swinholide A for 2 hours, and then exposed to 2 Gy irradiation. Representative IF images showing γH2AX foci in different groups of cells (scale bar = 5 μm). The number of γH2AX foci per cell was presented at the indicated time points following IR (data were generated from three independent experiments. * *p* < 0.05, ** *p* < 0.01).


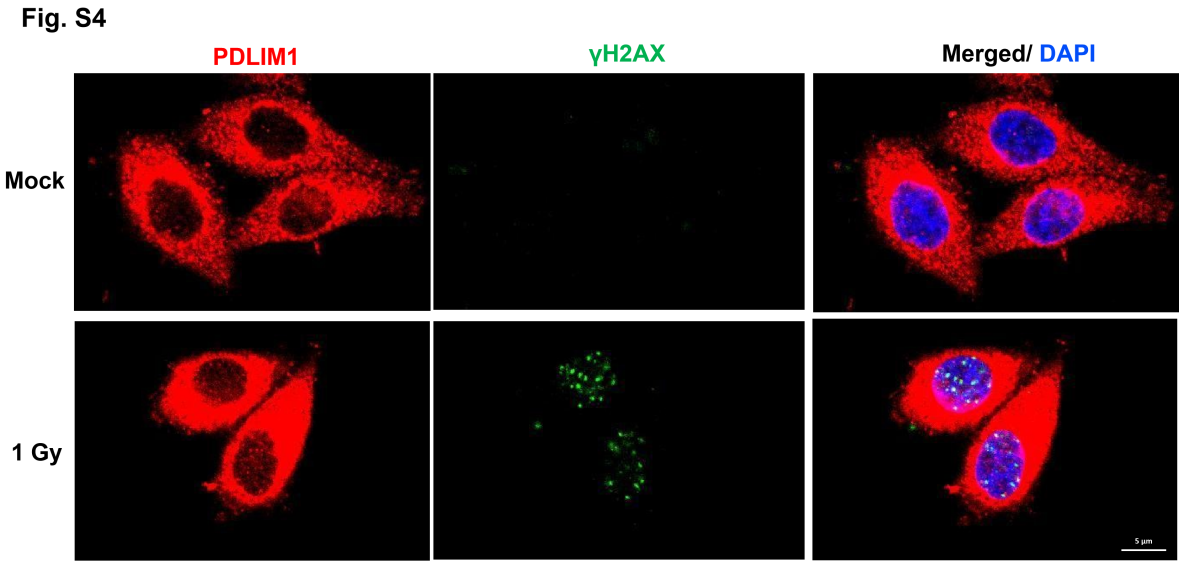


**Supplemental Figure 4.** The localization of PDLIM1 in HeLa cells after mock- or IR treatment..


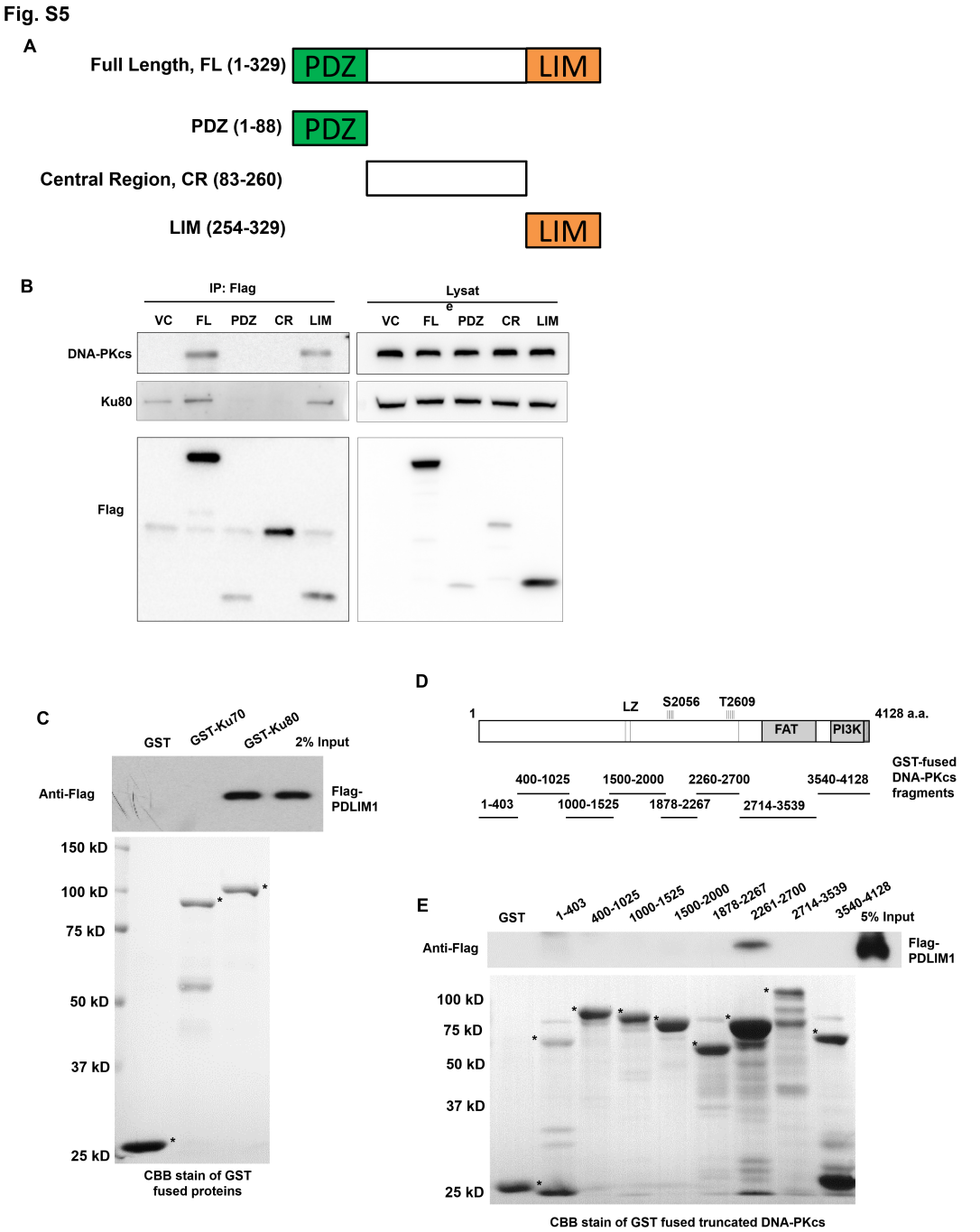


**Supplemental Figure 5.** (**A**) Schema of the truncated domains of PDLIM1. (**B**) Various cDNA constructs of PDLIM1 truncations were transfected into HeLa cells and immunoprecipitated using anti-Flag antibody; DNA-PKcs, Ku80 and Flag signal was examined by Western blotting. (**C**) GST, GST-fused Ku70 and Ku80 were incubated with purified Flag-PDLIM1 protein, the interaction between PDLIM1 with indicated proteins were detected by Western blotting. The GST, GST-fused Ku70 and Ku80 proteins were visualized by Coomassie brilliant blue (CBB) stain. (**D**) Schema of the truncated domains of DNA-PKcs. (**E**) GST and GST-fused DNA-PKcs truncated fragments were incubated with purified Flag-PDLIM1 protein, the interaction between PDLIM1 with indicated fragments were detected by Western blotting. The GST and GST-fused DNA-PKcs truncated fragments were visualized by Coomassie brilliant blue (CBB) stain.


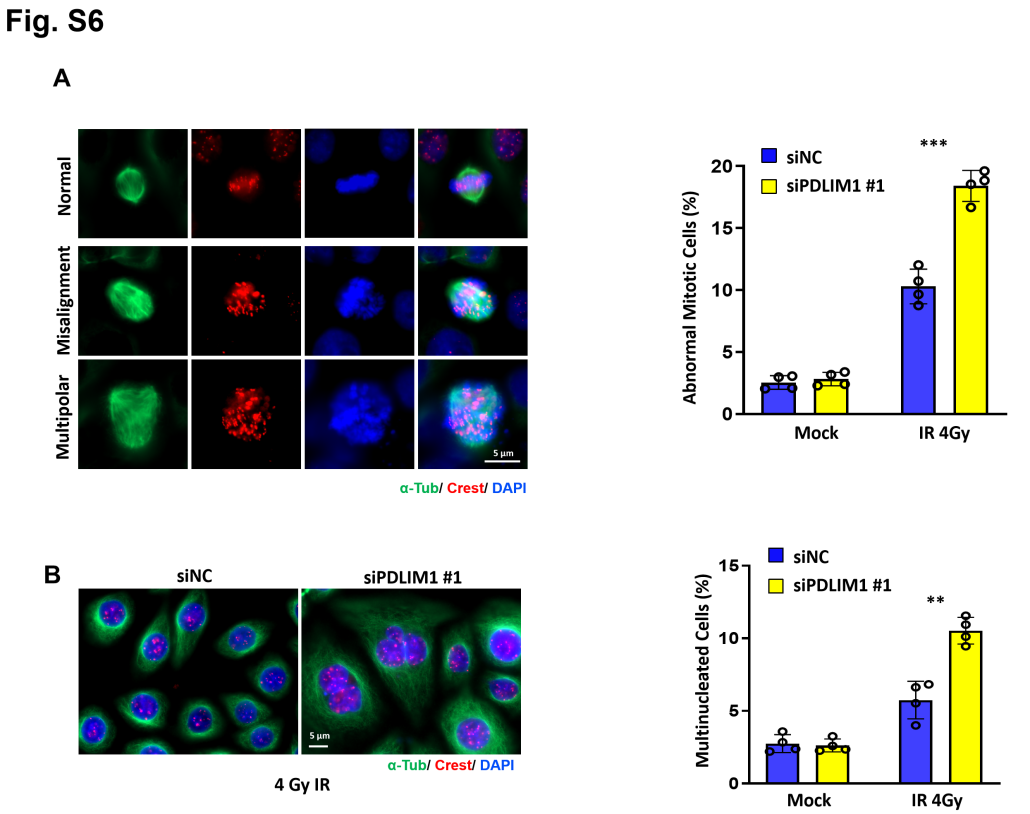


**Supplemental Figure 6.** Deficiency of PDLIM1 leads to increased outcome of aberrant spindles (**A**) and multinucleated cells in response to IR (**B**).
